# A study protocol to determine the association between lifetime lead exposure and violent criminal behaviour in young males in conflict with the law

**DOI:** 10.1186/s12889-019-7025-5

**Published:** 2019-07-11

**Authors:** Thokozani P. Mbonane, Angela Mathee, André Swart, Nisha Naicker

**Affiliations:** 1 Department of Environmental Health, Faculty of Health Sciences, University of Johannesburg, Johannesburg, South Africa; 2 Environment and Health Research Unit, South African Medical Research Council, Johannesburg, South Africa; 3 School of Public Health, Faculty of Health Sciences, University of the Witwatersrand, Johannesburg, South Africa; 4 The Epidemiology and Surveillance Section, National Institute for Occupational Health, National Health Laboratory Services, Johannesburg, 2000 South Africa

**Keywords:** Bone, Blood, Lifetime, Lead exposure, Violent, Criminal, Behaviour, X-ray fluorescence, Environmental health

## Abstract

**Background:**

Low-level lead exposure has harmful and persistent effects on behaviour. Recent studies have linked environmental lead exposure and the development of aggressive, violent and criminal behaviour. This protocol is designed to study an association between lifetime (bone) lead levels and violent criminal behaviour among young males in conflict with the law in Gauteng youth development centres.

**Methods:**

This paper describes a study to determine a link between lifetime lead exposure and violent criminal behaviour. Lifetime lead exposure will be measured using bone lead measurement, while blood lead levels will be observed for current exposure. Thereafter, criminal records of participants will be reviewed whereas violent behaviour and risk factors will be observed using a questionnaire. The study focused on young males in conflict with the law in three centres within Gauteng Provence, South Africa. After stratifying the centres, we randomly selected participants. The researcher shall adhere to ethical requirements throughout the study. Data will be analysed for descriptive and inferential analysis using Statistical Package for Social Science (SPSS).

**Discussion:**

The study will provide a strong foundation for an improved understanding of the relationship between environmental contamination from lead exposure and aggression/violent criminal behaviour. Beyond the health sector, the study findings may be able to inform new approaches to crime prevention through environmental action with an emphasis on the role of non-health sectors.

## Background

Environmental lead exposure at an early life is a growing important risk factor to societal ill-behaviour [1, 2]. A recent study estimates that about 400,000 deaths per annum are attributable to lifetime lead in develop countries [3]. Yet, African children still have blood lead levels above 5 μg/dL [4]. There is growing evidence that links long-term environmental lea d exposure with neurological effects, violent and criminal behaviour [5–9].

Elevated blood lead levels may disturb the cells’ biological metabolism through replacing useful ions in the body such as calcium, magnesium, iron and sodium. It also impairs or disrupts other biological processes in the human body [10, 11]. According to Flora et al. once calcium has been substitute by lead, the protein kinase C that regulates neural excitation and memory storage can be weakened [12]. This may lead to brain disorder resulting in reduced intelligent quotient, behavioural changes such as a shortened attention span, learning problems and increased antisocial behaviour such as violence and criminal acts [6, 13–15]. Ecological and epidemiological studies in developed countries have linked lead exposure to antisocial and criminal activities [16–22]. A retrospective cohort study conducted in the USA found that children with higher blood lead at birth were at risk of being arrested for a violent crime [23]. Similar findings were supported by studies conducted in other developed countries [7]. However, a study in New Zealand found that there is no causation relationship between higher blood levels and criminal activities [24].

South African children are still exposed to lead found in their living, playing and parents’ working environments [25, 26]. Little information is available in South Africa regarding the health effects of lead exposure [27], especially in adolescents, young adults in conflict with the law and specific high-risk groups. A recent study by Nkomo and colleagues found that males in early teens with elevated blood lead levels were at risk at perpetration violence behaviour in their late adolescence stage [5]. South African children still have blood lead levels above the Centers for Disease and Control and Prevention (CDC)'s recommended reference level of 5 μg/dL [28–31]. In addition South Africa, there is a high number of violent or aggressive crime such as murder, hijacking with weapons committed on a regular basis. Hence, it is important to understand the role of environmental lead exposure to criminal behaviour in developing countries (South Africa included). Therefore, this study aims to bridge the gap in the evidence of this risk in South Africa.

Based on evidence emerging from well-resourced countries [32], the current study hypothesizes that incarcerated young males in South Africa who have been charged or adjudicated for violent criminal acts have high bone and blood lead levels. The first objective of the study we determining and comparing bone and blood lead measurements in young males in conflict with the law in three youth development centres in Gauteng; secondly, determining and describing violent criminal behaviour and health status in the study population through the administration of a questionnaire that includes a behavioural assessment and lastly examine the association between lifetime (bone) and current (blood) lead levels and violent criminal behaviour among young males in conflict with the law in Gauteng youth development centres.

## Methods

### Study design and population

A cross-sectional analytical study was designed to determine the association between bone lead levels and violent criminal behaviour among South African young males in conflict with the law.

### Population and sampling

The study population consists of young males aged 14–21 (age group accommodated in the facilities) who have committed and been convicted of a crime (ranging from serious violence to less serious crimes). These young males are accommodated at three child and youth development centres (namely Centre A, B & C for the purpose for this study) in the Gauteng Province. Centre A houses a maximum of 50 young males convicted of serious crimes, Centre B provides beds for 100 young males that have committed less serious crimes, and Centre C accommodates 100 sentenced young males that have committed both serious and less serious crimes but have agreed to a diversion programme (rehabilitation services). We adopted a stratified random sampling technique to group the centres (centres were grouped according to the type of crime committed) and select participants. Each centre formed a stratum; thereafter participants are randomly sampled to avoid selection bias.

### Recruitment

Firstly, the researchers requested and received permission from the Gauteng Department of Social Development to access the centres. Secondly, we approach the parents or legal guardians of a male under the age of 17 for consent to approach their children to partake in the study. Lastly, after receiving parental consent, we approach all the young males in the centres and sort assent and consent.

### Sample size determination

The adequate sample size was calculated on the estimated population of 250 and prevalence of lead exposure and violent behaviour of 30% (CI: 95%), based on a study conducted amongst adolescents to determine the association between childhood lead exposure and violent behaviour [5]. We used the following equations to calculate for adequate sample size; where: n = minimum sample size; Z = standard normal deviate at α probability (1.96); P = Prevalence of BLLs and violent behaviour in South African male adolescents (30%); ME = margin of error (0.05) and CI = confidence level (95%).

### Data collection

Different methods and tools were designed and adopted for data collection. Data will be collected in four phases; record review, questionnaire, blood sample, anthropometric measurements and bone measurement. The research assistants comprise a phlebotomist/nurse for blood sampling, as well as taking and calculating the calculate body mass index (BMI), while an XRF certified technologist (for taking bone lead measurements). In the first phase, the records of young males in the centres were reviewed in order to obtain their names for the allocation of numbers and the type of crime committed.

#### In vivo bone lead measurement (tool 1)

Tibial bone lead concentration levels are estimated using non-invasive in vivo K-shell X-ray fluorescence (KXRF). The KXRF technique is a biomarker used to determine cumulative lead exposure levels [33–37]. Participants in the study will be required to sit while the measurement system is fitted. The tibia of the study participant will be exposed to X-ray fluorescence for 30 min. Participants’ exposure to radiation due to X-rays is approximately 2.1 μSv, which is less than the radiation experienced during chest X-rays [38]. Participants are expected to remain seated and still for this period. The participants do not experience any pain as the process is the same as for a normal X-ray. Once the readings (Resultant spectra) are captured, these will be sent to an expert from Mount Sinai University for analyses.

#### Blood sample (tool 2)

Venous blood samples (50–100 μl) are collected from participants using a sterile (free of trace metals) test tube (BD Vacutainer system) containing the anticoagulant ethylene diamine tetra acetic acid (EDTA) by registered nurses from the SAMRC. All samples are analysed using Inductively Coupled Plasma Mass Spectrometry (ICP-MS) for BLLs. A drop of the blood will be collected from the site where venous blood will be withdrawn, before applying pressure and plaster to assess for anaemia, to determine haemaglobin levels (since blood lead concentrations are affected by haemaglobin concentration).

#### Questionnaire (tool 3)

Information of the participants’ parents’ occupational history, socio-demographic, environmental issues, biological factors, and behavioural issues is ideally collected using a questionnaire. The questionnaire is translated by a competent person to isiZulu and seSotho for those who may not understand English.

### Data storage

We created a profile on the REDCap software application, an online (secure web-based) application for data storage and management. Thereafter, the tools are pre-programmed on the REDCap software application to capture the responses and readings. After capturing the data on the application and all the responses have been completed, the data will be kept in the application and a password will be created by the principal investigator.

### Quality control

The in vivo signal from a subject measured for each bone lead X-ray are analysed and compared to the established calibration line to obtain one or more estimates of the subject’s bone-lead level. We sought assistance from an expert in the field for guidance during data collection and analysis. Furthermore, a qualified agent calibrates the instrument prior to commencing with the study. Blood samples will be taken by a professional nurse or doctor, currently registered with the Health Professions Council of South Africa (HPCSA) or/and South African Nursing Council (SANC). Thereafter, refrigerated prior to being transported to an accredited laboratory, which conforms to international and national quality control standards [39]. The questionnaire was constructed and finalized with the assistance of the study supervisors and a biostatistician. We piloted the questionnaire in advance to identify concerns and institute refinements.

### Data management and analysis

Data from the questionnaire, bone measurements and BLLs readings are entered and captured on REDCap for storage, data cleaning, and identifying missing data. Thereafter, data transferred to SPSS statistical analysis programme for data analysis. A score is developed from the two behaviour checklists behavioural assessment section, type of crime committed will be classified according to serious or less crime and based on the established criteria will be categorised as indicating violent and criminal. Based on the behaviour checklists and criminal record established criteria will be categorised as having violent criminal behaviour or not (binary variable). Bone and blood lead distributions and criminal record information will be analysed for normality and presented using descriptive statistics (range, median, mean, standard deviation, 95% confidence intervals); while non-normally distributed variables will be log-transformed and analysed using parametric tests (Analysis of variance - ANOVA). The relationships between the dependent variables (bone and lead levels) and independent variables (socio-demographic, environmental and showing violent criminal behaviour) will be determined using multiple bivariate logistic regression techniques (will be presented in odds ratios). We will use a stepwise method, where a liberal criterion (*p*-value) of 0.2 will be used to select variables. Then a backward or maximum likelihood ratio test for variables inclusion criteria will be implemented to select variables significant at the 95% confidence interval for the multivariable regression to estimate the likelihood of having an outcome of interest (violent criminal behaviour). Furthermore, ANOVA and multivariate linear regression modelling will be used to compare mean blood and bone lead levels among the study participants of the three centres. Continuous variable (bone lead and blood lead) will be categorised, for example, blood lead (0 ≤ 5μd/dL and 1>5μd/dL) and bone lead (0 ≤ 25 ppm and 1 > 25 ppm). Pearson’s chi-square (χ2), Fisher’s exact, proportionality and two sample student t-test tests and ANOVA will be applied to determine the association among blood, bone lead levels, violent criminal behaviour and confounding variables.

### Outcomes

The primary outcome of this study is to describe the association between lifetime environmental lead exposure and violent criminal behaviour. Furthermore, to describe the difference in bone and blood lead levels in young males that are in the juvenile centre. After controlling for confounding factors such: age, parental educational levels, parents occupation, backyard business, socio-economy status, smoking, residential proximity to industries and drinking water from lead water-taps.

### Dissemination

We aim to disseminate the results from the study to the participants, policymakers in the health and criminal justice sector through presentations and official report. Furthermore, presents at relevant conferences and publish the findings in peer-reviewed publications.

### Study progress

The study has received ethical approval to commence with the project. All the lists of young males from different centres have been received and parents of males under the age of 18 years have been approach for consent. The researchers are currently busy with data collection (study ongoing).

## Discussion

Studies have shown that lead exposure remains high in developing countries, including South Africa [2, 30, 40]. Despite continued exposure to lead, especially in children of low socio-economic status, there is a dearth of information on the contribution of lead exposure to violent criminal behaviour in South Africa. This study is the first (according to our knowledge) study in South Africa to look at the association between violent criminal behaviour and lead in blood (acute exposure) and bone (a lifetime exposure) among young males in conflict with the law.

Regardless of the higher burden of lead exposure in South Africa and other countries, gaps still exist in the knowledge of lead exposure and its impact on adolescents and young adults. One of the strengths of this study is that it seeks to understand the lifetime lead exposure with violent criminal behaviour during late adolescence and young adulthood. Most of the studies have compared blood lead levels when they were children with an antisocial tendency at a later stage. These studies did not consider accumulative lead levels from long-term exposure, which exclude lifetime exposure lead index. This study will contribute to the knowledge gaps on the effect of lifetime lead exposure on antisocial behaviour in South Africa and internationally. In recent years few studies have been reported that associated blood lead levels with antisocial behaviour such as aggressive and violent behaviour in South Africa. In this study, we going to measure for both cumulative and recent exposure and compare these lead levels. This is believed to be more informative than looking at one type of exposure. Secondly, the study will use two different approaches to determine aggressive and criminal tendencies, which will ensure that the outcome of interest is verified and supported.

Neurobehavioral effects link to lead exposure are preventable and reversible. A prospective longitudinal study in Taiwan conducted amongst lead workers showed that the implementation of an intervention programme is essential to reduce blood lead levels has the potential to reverse neurobehavioural caused by lead exposure [41]. It is important to understand the role of lead exposure to violent and criminal tendencies, thus contributing to the fight against crime using environmental health intervention programmes.

The limitation of the study is that it seeks to identify an association between violent criminal behaviour that occurred before the participants were placed in the centres. Therefore, it would not be able to determine causation of the phenomena. Furthermore, the study will compare children in conflict with the law only and based on the crime committed using a cross-sectional study.

## Data Availability

Data sharing is not applicable to this article as no datasets were generated or analysed during the current study.

## Abbreviations

ANOVA: Analysis of variances
BLL: Blood lead levels
BMI: Body mass index
CDC: Centers for Disease Control and Prevention
EDTA: Ethylene diamine tetra acetic acid
HPCSA: Health Professional Council of South Africa
ICP-MS: Inductively coupled plasma mass spectrometry
KXRF: K-shell X-ray fluorescence
ME: Margin of error
ppm: parts per million
REC: Research ethics committee
SAMRC: South African Medical Research Council
SANC: South African Nursing Council
SSPS: Statistical Package for Social science
USA: United Nations of America
XRF: X-ray fluorescence
μg/dL: microgram per deciliter
μSv: microsieverts
